# Educational Inequalities in Acute Myocardial Infarction Incidence in Norway: A Nationwide Cohort Study

**DOI:** 10.1371/journal.pone.0106898

**Published:** 2014-09-04

**Authors:** Jannicke Igland, Stein Emil Vollset, Ottar K. Nygård, Gerhard Sulo, Marta Ebbing, Grethe S. Tell

**Affiliations:** 1 Department of Global Public Health and Primary Care, University of Bergen, Bergen, Norway; 2 Department of Health Registries, Norwegian Institute of Public Health, Bergen, Norway; 3 Section for Cardiology, Department of Clinical Science, University of Bergen, Bergen, Norway; 4 Department of Heart Disease, Haukeland University Hospital, Bergen, Norway; Scuola Superiore Sant'Anna, Italy

## Abstract

**Background:**

Increasing differences in cardiovascular disease (CVD) mortality across levels of education have been reported in Norway. The aim of the study was to investigate educational inequalities in acute myocardial infarction (AMI) incidence and whether such inequalities have changed during the past decade using a nationwide longitudinal study design.

**Methods:**

Data on 141 332 incident (first) AMIs in Norway during 2001–2009 were obtained through the Cardiovascular Disease in Norway (CVDNOR) project. Educational inequalities in AMI incidence were assessed in terms of age-standardised incidence rates stratified on educational level, incidence rate ratios (IRR), relative index of inequality (RII) and slope index of inequality (SII). All calculations were conducted in four gender and age strata: Men and women aged 35–69 and 70–94 years.

**Results:**

AMI Incidence rates decreased during 2001–2009 for all educational levels except in women aged 35–69 among whom only those with basic education had a significant decrease. In all gender and age groups; those with the highest educational level had the lowest rates. The strongest relative difference was found among women aged 35–69, with IRR (95% CI) for basic versus tertiary education 3.04 (2.85–3.24)) and RII (95% CI) equal to 4.36 (4.03–4.71). The relative differences did not change during 2001–2009 in any of the four gender and age groups, but absolute inequalities measured as SII decreased among the oldest men and women.

**Conclusions:**

There are substantial educational inequalities in AMI incidence in Norway, especially for women aged 35–69. Relative inequalities did not change from 2001 to 2009.

## Introduction

Educational inequalities in cardiovascular disease (CVD) and coronary heart disease (CHD) mortality rates have been reported for the last decades,[1], [2] also in Norway, where inequalities have increased.[3], [4] CHD mortality rates have declined substantially over the last decades,[5] partly because of improved survival after acute myocardial infarction (AMI).[6]–[8] In addition, previous studies have reported different survival rates according to educational levels and it is not clear whether the improvement in survival has occurred in all educational levels.[9]–[11] Thus, true inequalities in the burden of disease may be better reflected using AMI incidence instead of mortality as the outcome.

Although educational inequalities in AMI incidence have been investigated,[11]–[15] most studies are either case-control studies[14] or small or medium-sized cohort-studies[16]–[18] with varying participation rates. We were able to identify six studies[11], [15], [19]–[22] on AMI incidence and educational level which covered the total population in a geographical area. Among these, only two were nationwide.[15], [22] Also, only two of the studies on educational inequalities in AMI-incidence reported whether the inequalities changed over time.[11], [22] In addition, in all studies on AMI incidence, only relative measures of educational inequalities are reported. From a public health perspective absolute educational inequalities in AMI incidence are also important.

The research project Cardiovascular Disease in Norway (CVDNOR) offers the opportunity to study nationwide trends in CVD in Norway.[23], [24] Using data from CVDNOR, we have recently reported decreasing incidence of AMI from 2001 to 2009, driven by a decrease in persons aged 45 and above, while no decrease was seen in persons younger than 45 years of age.[25]

The present study expands this previous report by examining educational inequalities in AMI incidence in Norway between 2001 and 2009 with special emphasis on differences between men and women and between different age groups. Both absolute inequalities and relative inequalities are reported. We also investigate whether the inequalities changed between 2001 and 2009.

## Methods

### Cardiovascular disease in Norway – CVDNOR

Cardiovascular disease in Norway (CVDNOR) is a collaborative project between the University of Bergen and the Norwegian Knowledge Centre for the Health Services. Details on the project and data collection have been reported previously.[23], [24] Briefly, all CVD hospitalisations between 1994 and 2009 were collected retrospectively from the patient administrative systems (PAS) at all somatic hospitals in Norway. Also, in order to include out-of-hospital deaths, information on all deaths during 1994–2009 where CVD was mentioned on the Death Certificate as underlying or contributing cause of death was retrieved from the Norwegian Cause of Death Registry. Hospitalisation data and mortality data were linked to the National Education Database (NEDB) using the personal identification number unique for each resident in Norway. In addition, data on the total population of Norway were retrieved from the Population Registry and linked to NEDB in order to get an education-stratified population-at-risk in 5-year gender- and age groups to be used in calculation of incidence rates.

### The population at risk

In the total Norwegian population, an AMI-free cohort for a given calendar year was defined as all persons 35–94 years of age living in Norway on Januar 1^st^ that year with no AMI-hospitalisations the previous seven years, i.e. the population at risk of getting an incident AMI. Persons aged <35 years and ≥95 years were excluded in order to make sure that most cases had finished their education at the time of event and because we expect the validity of hospital discharge diagnoses, causes of death and information on education to be lower in very old persons.

### Outcome - Incident acute myocardial infarction

The outcome was incident AMI, defined as a hospitalisation with AMI (ICD9: 410, ICD 10: I21, I22) as main or secondary diagnosis or death with CHD (ICD9: 410–414, ICD10: I20–I25) as underlying cause without any AMI-hospitalisations during the seven year period before the event.[25] We identified 144 634 incident AMI cases aged 35–94 years during 2001–2009. We excluded 3302 cases because of invalid or missing data, resulting in inclusion of 141 332 incident AMIs.

### Main exposure - Level of education

AMI cases and the population at risk were categorized according to the highest completed educational level registered in NEDB the year before the incident AMI.NEDB contains data on the highest achieved education for all persons with a permanent address in Norway.[26] For persons who completed their education before 1970, this is based on self-reported education from the census in 1970, while information on education achieved after 1970 is based on yearly reporting from educational institutions to Statistics Norway. The highest achieved education is coded according to the Norwegian Standard Classification of education with level codes from 0 to 8, where 0 means no or only pre-school education and 8 means doctoral degree level. We categorized education levels into three categories: Basic education (compulsory education), upper secondary education (high school or vocational school) and tertiary education (college or university).

### Statistical analyses

Gender differences in age at the time of the incident AMI were tested using t-test and in the distribution of education using chi-square tests.

Incidence rates per calendar year were calculated using number of incident AMIs and the AMI-free population in 5-year age groups. Direct age-standardisation was done using the total Norwegian population in the year 2001 as standard population. Ninety-five percent confidence intervals (CI) for the age standardised rates were calculated using a normal approximation to the binomial distribution.

Calculations were done separately for each level of education in four groups; men and women 35–69 and 70–94 years.

Log-linear time-trends in incidence rates for each level of education were tested by including calendar year as a continuous independent variable in a Poisson regression model with count of AMI-events as outcome and the AMI-free population specified as exposure. Models were constructed for each gender- and age group separately with adjustment for age.

Incidence rate ratios (IRR) with tertiary education as the reference category were also calculated with Poisson regression. Separate models were made for the four age and gender-groups with adjustment for age and calendar year. Potential change in the effect of education over time was explored by stratifying on calendar year for each gender- and age group and by testing for interaction between calendar year and education in age-adjusted models.

During the study period (2001–2009), level of education among adults were strongly associated with both age and gender in Norway with lower education among the elderly and also lower education among women compared to men in the oldest age groups.[27] In addition, the proportion in the population with tertiary education increased slightly between 2001 and 2009, especially among younger women.[28] The distribution of education for the population in Norway during 2001–2009 according to gender and age group (35–69 and 70–94) are displayed in Figure S1. Therefore, in order to compare the effect of education on incidence across age groups, gender and calendar years we also calculated the relative index of inequality (RII) and the slope index of inequality (SII) for each gender and age group.[29], [30] Each individual were given an education risk score between 0 and 1 which was equal to the midpoint of the cumulative relative frequency range covered by the education distribution for each calendar year. Individuals with low education and thus higher risk of AMI had risk scores close to 1.

After calculation of the education risk scores, the RII was calculated as exp(β) from a Poisson model with the scores included as continuous variables. The SII was then calculated as the difference between predicted incidence rates when score was equal to 1 and 0 while keeping the other covariates in the model at the mean, using the margins-command in Stata.[31]

The RII is the ratio in rates between the 100^th^ and the 0^th^ percentile of the education distribution while SII is the difference in rates between the 100^th^ and the 0^th^ percentile of the education distribution.

A test for linear trend in RIIs and SIIs across calendar years was done with weighted linear regression with the RIIs and SIIs as dependent variables, calendar year as independent continuous variable and the inverse of the standard errors for the RIIs and SIIs as weights.

In our definition of incident AMI we included all CHD deaths outside hospital without any prior AMI hospitalisations the previous seven years. This could cause an overestimation of the AMI incidence because some of the CHD-deaths could have other causes than AMI. We therefore did additional analyses where only deaths with AMI as the underlying cause were included in order to see if this affected the findings of educational inequalities. The results of these analyses are included as supporting material.

All analyses were conducted using Stata 12 (Stata Corp LP, 4905 Lakeway Drive, College Station, Texas, USA).

The study protocol was approved by the Regional Committee for Medical and Health Research Ethics, Health Region West.

## Results

Among the 141 332 incident AMIs, 111 993 were hospitalised cases and 29 339 were CHD-deaths without prior AMI (Table 1). Women were on average older than men at their incident AMI and had less education. Patients with CHD-death without AMI hospitalisation were older and had lower education than patients with AMI-hospitalisation as the incident event.

**Table 1:** Incident AMIs in Norway 2001–2009 by level of education: a CVDNOR project.

	Total	Men	Women	p-diff
Total incident AMIs*				
n (%)	141 332 (100)	84 225 (59.6)	57 107 (40.4)	
Age, mean (SD)	73.7 (13.2)	70.4 (13.3)	78.6 (11.4)	<0.0001
Education, n (%)				
Basic education	69 203 (49.0)	35054 (41.6)	34149 (59.8)	
Upper Secondary education	57 507 (40.7)	38073(45.2)	19434 (34.0)	
Tertiary education	14 622 (10.4)	11098 (13.2)	3524 (6.2)	<0.0001
Hospitalised incident AMIs				
n (%)	111 993	68 154 (60.9)	43 839 (39.1)	
Age, mean (SD)	72.4 (13.3)	69.3 (13.3)	77.3 (11.7)	<0.0001
Education, n (%)				
Basic education	52 948 (47.3)	27 349 (40.1)	25 599 (58.4)	
Upper Secondary education	46 710 (41.7)	31 373 (46.0)	15 337 (5.0)	
Tertiary education	12 335 (11.0)	9 432 (13.8)	2 903 (6.6)	<0.0001
CHD**-deaths without prior AMI				
n (%)	29 339	16071 (54.8)	13268 (45.2)	
Age, mean (SD)	78.5 (11.7)	74.9 (12.3)	82.9 (9.2)	<0.0001
Education, n (%)				
Basic education	16255 (55.4)	7705 (47.9)	8550 (64.4)	
Upper Secondary education	10797 (36.8)	6700 (41.7)	4097 (30.9)	
Tertiary education	2287 (7.8)	1666 (10.4)	621 (4.7)	<0.0001

*AMI  =  Acute myocardial infarction;

**CHD  =  Coronary Heart Disease.

**Figure 1:**
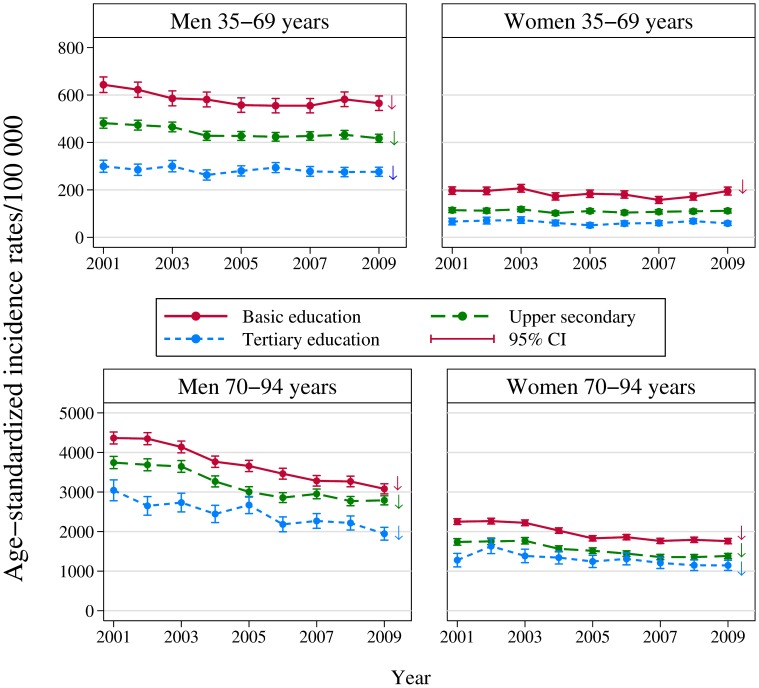
Time-trends in age-standardised AMI incidence rates by level of education: a CVDNOR project. Rates are shown in four gender- and age groups. Upper panel: Men and women aged 35–69 years. Lower panel: Men and women aged 70–94 years. Arrows indicate significant log-linear trend.

Time-trends in age-standardized incidence-rates for each level of education in the four gender- and age groups are shown in Figure 1. All four groups showed a clear education gradient with higher incidence rates for lower levels of education. The rates decreased significantly over time (log-linear time trend) for all levels of education except for among women aged 35–69 with upper secondary and tertiary education. Women aged 35–69 with basic education had a significant negative log-linear trend between 2001 and 2007 but rates increased on average by 11% per year from 2007 to 2009 (IRR (95% CI) = 1.11 (1.04–1.18)). Tests for interaction between year and education showed no proof of different log-linear trends across different levels of education in any of the four gender-and age-groups.

IRR for basic and secondary vs. tertiary education, RII and SII are given in Table 2. IRRs were significant in all gender- and age groups. The strongest effect was among women aged 35–69 with an IRR (95% CI) of 3.04 (2.85–3.24). Poisson models with test for interaction between education and year did not show a significant change in the education-effect over time in any of the four gender- and age groups. The RII was strongest among women aged 35–69 with RII (95% CI) of 4.36 (4.03–4.71). SII was smallest among women aged 35–69 and largest among men aged 70–94.

**Table 2:** Relative and absolute inequalities in AMI incidence according to level of education by gender and age group: a CVDNOR project.

	Total	Men 35–69	Women 35–69	Men 70–94	Women 70–94
Incident AMIs	141 332	37 031	10 886	47 194	46 221
Person-years 2001–2009*	21 037 794	8 782 941	8 782 962	1 543 750	2 428 438
AASIR 2001–2009	715.5	436.2	122.0	3299.6	1757.7
IRR (95% CI)					
Tertiary education	1	1	1	1	1
Upper secondary education	1.44 (1.41–1.47)	1.56 (1.52–1.61)	1.86 (1.74–1.98)	1.32 (1.28–1.36)	1.20 (1.15–1.25)
Basic education	1.82 (1.78–1.85)	2.01 (1.95–2.07)	3.04 (2.85–3.24)	1.54 (1.49–1.59)	1.52 (1.46–1.58)
p-trend	<0.001	<0.001	<0.001	<0.001	<0.001
RII (95% CI)	2.10 (2.06–2.15)	2.45 (2.35–2.55)	4.36 (4.03–4.71)	1.64 (1.59–1.70)	1.72 (1.66–1.79)
SII (95% CI)	462.7 (449.1–476.3)	336.3 (320.8–351.8)	138.1 (129.8–146.3)	1953.2 (1815.8–2090.6)	1150.6 (1070.3–1231.0)

Abbreviations:

AMI, Acute myocardial infarction.

AASIR, Average age-standardised incident rate between 2001 and 2009.

IRR, Incidence rate ratio from Poisson regression.

RII, relative index of inequality. Ratio between rates at the upper 100th- and lower 0^th^ %-end of the education scale.

SII = slope index of inequality, absolute difference in rate per 100 000 between the upper 100^th^ %- and lower 0^th^ %-end of the education scale.

IRR, RII and SII are adjusted for age and calendar year in each gender- and age strata. Total model also adjusted for gender.

*Person-years for the total population at risk of getting an incident AMI.

**Figure 2:**
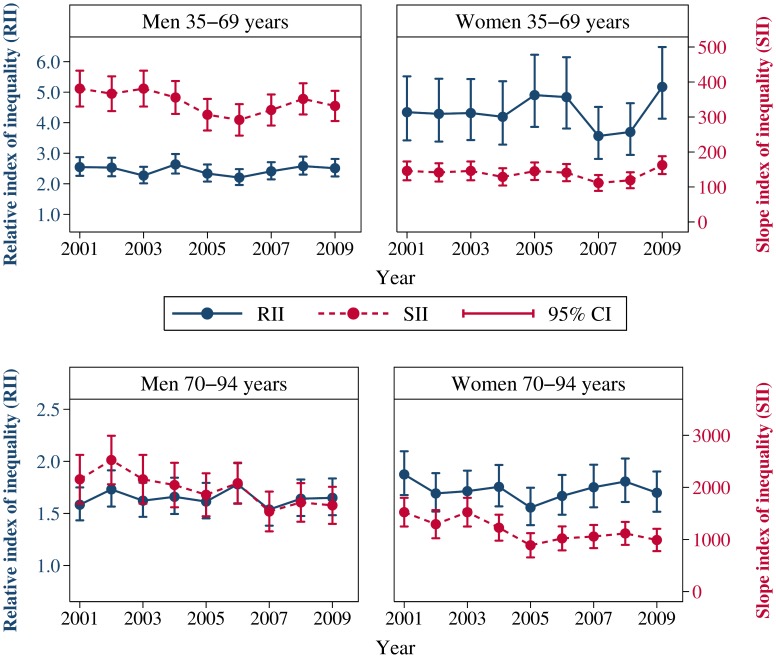
Time-trends in relative index of inequality (RII) and slope index of inequality (SII): a CVDNOR project. Estimates are displayed by gender and age group. Upper panel: Men and women aged 35–69 years. Lower panel: Men and women aged 70–94 years. The y- axis for the RII is given on the left hand side and the y-axis for the SII is given on the right hand side.

RIIs and SIIs from analyses stratified on calendar year are given in Figure 2. There were no linear trends in RIIs over time in any of the gender and age groups. Among men and women 35–69 years, the SIIs showed no linear trend, while among men and women 70–94 years there was a statistically significant linear decrease with p-trend = 0.02 among men and p-trend = 0.006 among women.

Results from additional analyses with only AMI-deaths included instead of all CHD-deaths are given in Table S1. The relative inequalities, measured ass IRR and RII, were very similar to the main results in Table 2 while the absolute inequalities were smaller as a consequence of lower incidence rates for all levels of education.

## Discussion

This nation-wide study in Norway showed substantial educational differences in AMI incidence rates. The relative inequalities were largest among women aged 35–69 years old, while the absolute inequalities were largest among men aged 70–94. The relative inequalities did not change during 2001–2009 while absolute inequalities decreased among the oldest men and women, due to the general decrease in incidence for all levels of education during 2001–2009.

### Comparison with other studies

A study on educational differences in first-time AMI in Sweden between 1987 and 2008 reported an IRR for less than 9 vs. more than 12 years of education to be 1.60 for men and 1.85 for women.[15] This corresponds well with the IRRs we found for upper secondary vs. basic education. A Finnish study on socioeconomic inequalities in acute coronary syndromes between 1988 and 2002 found significant differences in age-standardized incidence rates between secondary and basic education which decreased with age, and stronger relative differences among younger women than younger men.[11] In accordance with our findings, they found no significant interaction between level of education and calendar year.

We are not aware of studies that have reported RII and SII for educational inequalities in AMI incidence, only CHD and CVD mortality.[1]–[3] Our estimate of RII in men aged 35–69 (RII = 2.45) is similar to the RII for CVD mortality in Norwegian men aged 46–64 between 1990 and 2000,[3] while the RII we found in women aged 35–69 (RII = 4.36) is somewhat higher than the corresponding RII for inequality reported for CVD mortality in women (RII = 3.7). This indicates that in women, the inequalities in the actual burden of disease are larger than the impression we get from studying mortality rates. Another study by Gallo et al based on data from the multi-center EPIC cohort-study found similar educational inequalities in men and women with a RII of 2.3 for men and 2.4 for women,[2] but the most socially deprived women may decline to participate, affecting the estimate of educational inequality in CHD mortality.

### Strengths and limitations

The CVDNOR study encompasses the total population, minimizing selection bias. By using the AMI-free population as population at risk instead of the total population we have also avoided an overestimation of incidence rates in the oldest group were a substantial proportion of the population have had an AMI. Emigration was taken into account by updating the population at risk January 1^st^ each year. All deaths among Norwegian residents are registered in the Cause of Death Registry, even if the death occurs abroad. Thus, the only loss to follow-up was non-fatal incident AMI hospitalisation outside Norway among non-emigrated residents.

While other studies on inequalities in health mostly reports relative measures,[32] we have reported both relative and absolute measures of inequalities according to recommendations from the WHO.[33] Relative and absolute measures of inequality provides different aspects of information and it is important to report both measures in order to get a broader picture on the inequalities in the burden of the disease. For instance, if the disease occurrence is decreasing over time for all educational levels but more for the most educated, the absolute inequalities will decrease, while the relative inequalities will increase. When only one measure is reported, the conclusion may be misleading and misinterpreted. We have also taken into account changes in the distribution of education in the population by reporting RII and SII instead of merely rate ratios and rate differences between categories of education.

By using education instead of occupational class, income or wealth as a measure of socioeconomic status (SES) we have also minimized the likelihood of reversed causation because education usually is completed early in life, before clinical CVD.[34] Low occupational class/income/wealth at the time of the event may be caused by many years of poor health status before the clinical event. Education is a precise and stable measure which, in Norway, is easier to obtain than other SES-measures. In addition, the different SES-measures do not measure the same aspects of socioeconomic status,[35] and education is perhaps more related to lifestyle choices and health awareness than income and wealth.

The identification of incident AMI cases in the study was based on diagnosis codes from the patient administrative systems without validation against the patient's medical records, but another project who used the same method to withdraw data on AMI-hospitalisations from the patient administrative systems in Norway found that less than 1% of the cases deviated from the patient medical record when it came to main diagnosis and dates for hospitalisation and discharge.[36] The troponin diagnostic criteria for AMI, which causes more AMIs to be diagnosed was introduced gradually in Norway between 1999 and 2001 and is thus not likely to have affected the trends in AMI incidence rates between 2001 and 2009.[37]

For non-hospitalised cases we included all CHD-deaths in the main results, which may overestimate the AMI incidence. Additional analyses with a stricter definition with only AMI-deaths included resulted in approximately the same relative educational inequalities. The potential overestimation of AMI incidence has thus not introduced any bias in the results, since unchanged relative inequalities imply that overestimation has occurred to the same extent for all educational levels.

Because of the self-report of education in those who completed their education before 1970, there is a risk of underreporting of education in the age group 70–94 which could have attenuated the education effect.

Further, formal education might not be a good enough measure of socioeconomic status among the elderly, since it was less common to complete higher education in Norway fifty years ago. Many elderly people, especially elderly women, with high socioeconomic status may nevertheless not have completed a high level of education. This probably explains the weaker relative inequalities we found among the oldest men and women compared to the youngest.

### Explanations and interpretation of results

The majority of the educational inequalities in AMI incidence may be explained by educational differences in CVD risk factor levels and health behaviour. Since our study is based on the total population of Norway and information on the cases was obtained from patient administrative systems at the hospitals, we were not able to adjust for CVD risk factors or other lifestyle factors. Statistics Norway delivers reports on health, life style and living conditions every third year based on results from telephone interviews and questionnaires in a representative sample of Norwegian residents.[38] According to these reports there are substantial educational inequalities in the prevalence of smoking, obesity and physical inactivity. The prevalence of smoking and physical inactivity decreased between 2002 and 2012 for all levels of education and age groups, while obesity increased. The relative educational inequalities in smoking prevalence also increased in all age groups in the same period while educational inequalities in obesity increased only among persons aged 25–44. A recent health survey from one of the 19 counties in Norway found substantial educational inequalities in smoking, hypertension and diabetes among both men and women.[39] Differences in smoking and hypertension increased between 1984–86 and 2006–08. The investigators also found that 25% of the educational inequalities in CHD mortality in women and 55% in men could be explained by behavioural factors including smoking, physical activity and alcohol intake.[40] We did not observe increasing educational inequalities in AMI incidence during 2001–2009, despite reports on increasing inequalities in risk factors during the same time period. However, since there normally is a time lag between poor lifestyle and occurrence of disease it might take some time before increasing inequalities in risk factors are reflected in incidence rates.

Some of the educational inequalities in AMI incidence might also be explained by differences in health awareness,[41], [42] psychosocial factors[43], [44] or differences in compliance to treatment and adherence to advices on change of lifestyle.[45]

The strongest relative educational inequalities were observed among women aged 35–69. This was also the only gender-and age group without a significant decrease in incidence for all levels of education during the study period. Only persons with basic education had a significant decrease, but with a worrying increase the last few years. Further investigations should be done to investigate if this unfavourable trend is continuing for women in this age group after 2009.

Norway has a history of strong egalitarian policies with equal access to both education and healthcare for all residents. Despite this, our study shows that relative educational inequalities in AMI incidence are fairly large and not decreasing. Even though incidence rates decreased for all educational levels, primary prevention efforts are needed to reduce the gap in incidence between persons with high and low education.

## Conclusion

AMI incidence rates decreased between 2001 and 2009 for all educational levels except among women 35–69 years with upper secondary and tertiary education. AMI incidence rates between 2001 and 2009 show a steep gradient across levels of education, especially for women aged 35–69. The relative educational differences did not change over time while the absolute inequalities decreased in men and women aged 70–94.

## Supporting Information

Figure S1 **Distribution of education in the Norwegian population from 2001 to 2009 by sex, age group and calendar year.**
(PDF)

Table S1 **Relative and absolute inequalities in AMI incidence according to level of education by gender and age group when using an alternative definition of incident AMI cases: a CVDNOR project.**
(DOCX)
