# Supplementary material for: Educational Inequalities in Acute Myocardial Infarction Incidence in Norway: A Nationwide Cohort Study
Source: PLoS One. 2014 Sep 4;9(9):e106898. doi: 10.1371/journal.pone.0106898 (PMC4154768; doi:10.1371/journal.pone.0106898)
Supplement: Figure S1 — Distribution of education in the Norwegian population from 2001 to 2009 by sex, age group and calendar year. (PDF) [file pone.0106898.s001.pdf]

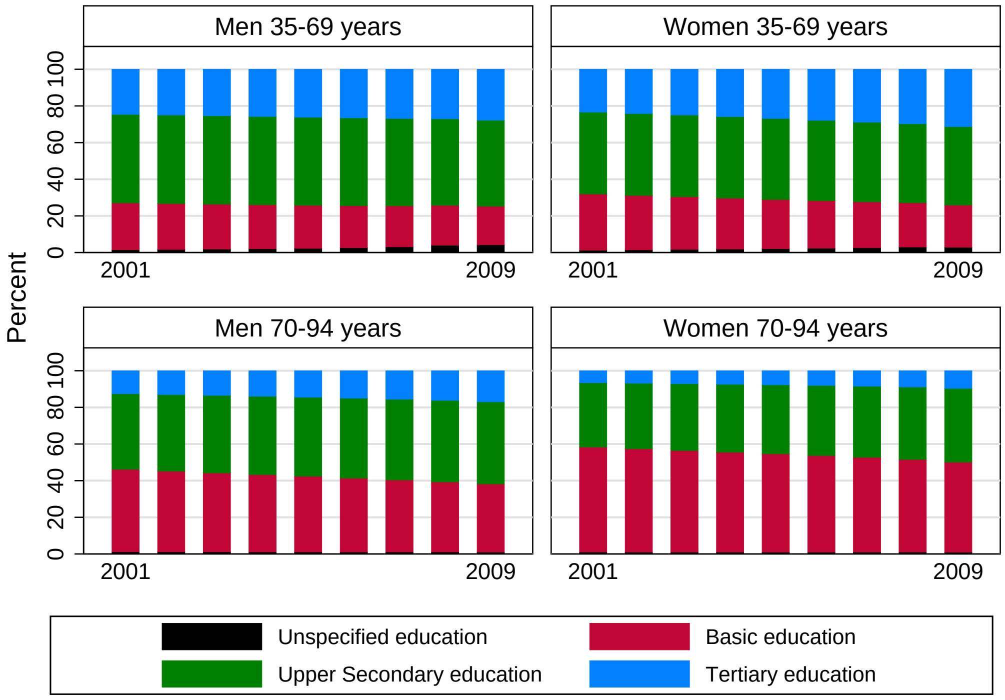

Men 35-69 years
Women 35-69 years
Men 70-94 years
Women 70-94 years
Percent
0
20
40
60
80
100
2001
2009
Unspecified education
Basic education
Upper Secondary education
Tertiary education
