# Supplementary material for: Educational Inequalities in Acute Myocardial Infarction Incidence in Norway: A Nationwide Cohort Study
Source: PLoS One. 2014 Sep 4;9(9):e106898. doi: 10.1371/journal.pone.0106898 (PMC4154768; doi:10.1371/journal.pone.0106898)
Supplement: Table S1 — Relative and absolute inequalities in AMI incidence according to level of education by gender and age group when using an alternative definition of incident AMI cases: a CVDNOR project. (DOCX) [file pone.0106898.s002.docx]

Supporting Table S1: Relative and absolute inequalities in AMI incidence according to level of education by gender and age group when using an alternative definition of incident AMI cases : a CVDNOR project.

|  | | Total | Men 35-69 | Women 35-69 | Men 70-94 | Women 70-94 |
| --- | --- | --- | --- | --- | --- | --- |
| Incident AMIs* | | 128 702 | 35 264 | 10 506 | 42 150 | 40 782 |
| Person-years 2001-2009** | | 21 037 794 | 8 782 941 | 8 782 962 | 1 543 750 | 2 428 438 |
| AASIR 2001-2009 | | 650.5 | 415.2 | 117.8 | 2931.5 | 1562.7 |
| IRR (95% CI) | |  |  |  |  |  |
|  | Tertiary education | 1 (ref) | 1 (ref) | 1 (ref) | 1 (ref) | 1 (ref) |
|  | Upper secondary education | 1.44 (1.41-1.47) | 1.55 (1.51-1.60) | 1.83 (1.72-1.96) | 1.30 (1.47-1.57) | 1.20 (1.15-1.26) |
|  | Basic education | 1.81 (1.77-1.84) | 1.98 (1.91-2.04) | 2.97 (2.78-3.18) | 1.52 (1.47-1.57) | 1.51 (1.44-1.58) |
|  | p-trend | <0.001 | <0.001 | <0.001 | <0.001 | <0.001 |
| RII (95 % CI) | | 2.08 (2.03-2.13) | 2.40 (2.30-2.49) | 4.24 (3.92-4.59) | 1.62(1.56-1.68) | 1.70 (1.63-1.77) |
| SII (95 % CI) | | 427.9 (414.6-441.1) | 314.6 (299.4-329.7) | 131.3 (123.2-139.4) | 1678.3 (1549.6-1806.9) | 995.9 (920.0-1071.8) |

Abbreviations:
AMI, Acute myocardial infarction
AASIR, Average age-standardized incident rate per 100 000 between 2001 and 2009
IRR, Incidence rate ratio from Poisson regression
RII, relative index of inequality. Ratio between rates at the upper 100th- and lower 0^th^ %-end of the education scale.
SII=slope index of inequality, absolute difference in rate per 100 000 between the upper 100^th^ %- and lower 0^th^ %-end of the education scale
IRR, RII and SII are adjusted for age and calendar year in each gender- and age strata. Total model also adjusted for gender.

*Incident AMI defined as hospitalisation with AMI as main or secondary diagnosis or death with AMI as the underlying cause of death without any AMI-hospitalisations the previous seven years.

**Person-years for the total population at risk of getting an incident AMI.
